# Supplementary material for: Rupture Forces among Human Blood Platelets at different Degrees of Activation
Source: Sci Rep. 2016 May 5;6:25402. doi: 10.1038/srep25402 (PMC4857101; doi:10.1038/srep25402)
Supplement: Supplementary Information [file srep25402-s1.pdf]

Supplementary Information

Rupture Forces among Human Blood Platelets at different Degrees of Activation

Thi-Huong Nguyen,[1,2,*] Raghavendra Palankar,[1,2] Van-Chien Bui,[1,2] Nikolay Medvedev,[1] Andreas Greinacher,[2*] and Mihaela Delcea[1,2,*]

[1]*Nanostructure Group, ZIK HIKE - Center for Innovation Competence, Humoral Immune Reactions in Cardiovascular Diseases, University of Greifswald, 17489 Greifswald, Germany.*

[2]*Institute for Immunology and Transfusion Medicine, University Medicine Greifswald, 17475 Greifswald, Germany*

E-mail: thihuong.nguyen@uni-greifswald.de; greinach@uni-greifswald.de; delceam@uni-greifswald.de

Supplementary Figure 1. SEM images of platelets on material-pasivated substrates at different surface-contact time.

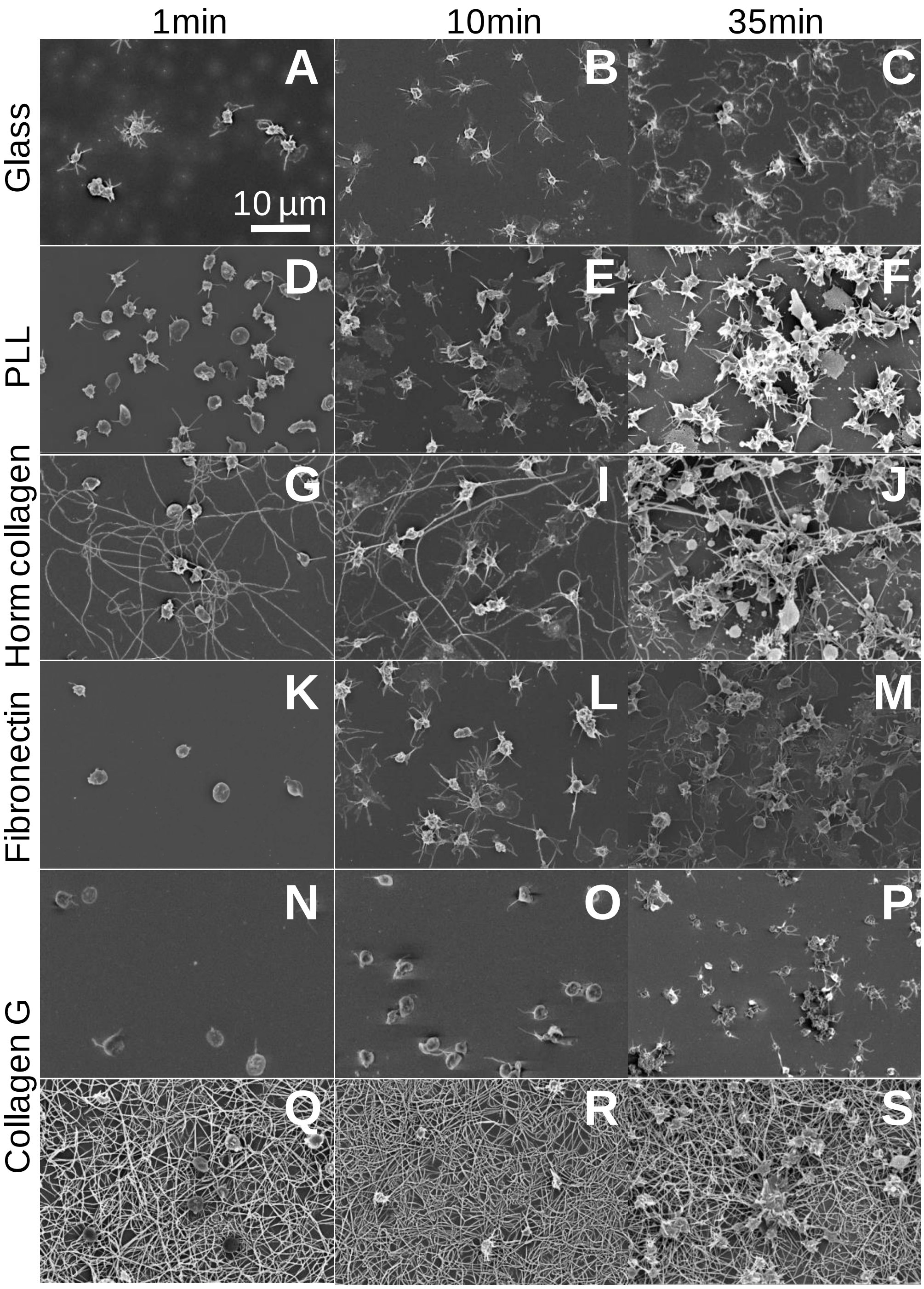

At each time point, platelets spread strongest on glass (**A-C**), PLL (**D-F**), and Horm collagen (**G-J**), weaker on fibronectin (**K-M**) and weakest on collagen G (**N-S**). At lower surface roughness (**N-P**), platelets seem to be spreading less on collagen G than that at higher surface roughness (**Q-S**). Thin filopodial extensions and irregular shapes are characteristics of activated platelets. Scale bar corresponds to 10 µm for all images.

Supplementary Figure 2. Platelet aggregation induced by different materials.

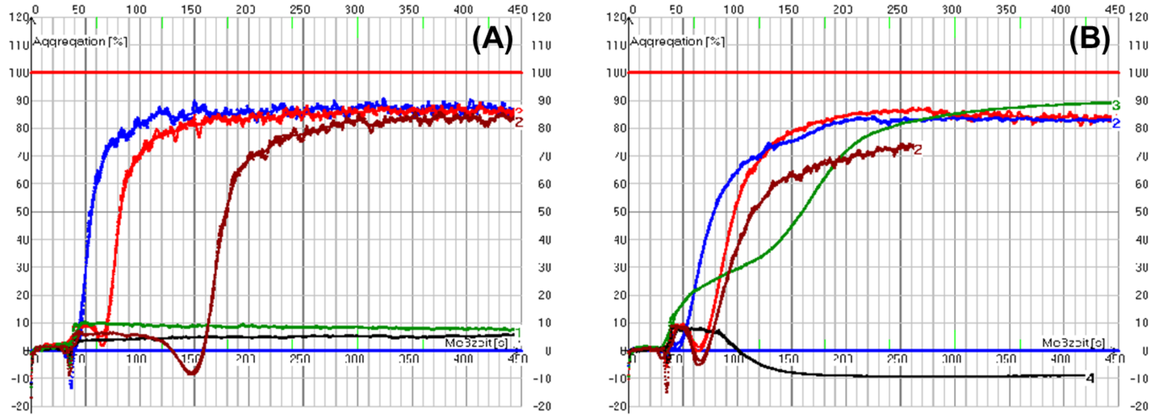

	Platelet-rich plasma			Washed platelets		
	Max aggregation [%]	Slope [%/min]	Lag Phase [s]	Maxaggregation [%]	Slope [%/min]	Lag Phase [s]
■ TRAP, 20 µg/ml	88.55	211.17	37.2	86.85	112.84	68.2
■ Horm Collagen, 5 µg/ml	87.14	187.58	68.0	83.65	108.85	53.2
■ PLL	9.94	38.70	10.0	89.63	56.43	34.2
■ Collagen G, 5 µg/ml	6.29	16.01	35.0	8.06	34.54	33.8
■ Collagen G, 100 µg/ml	84.97	156.53	157.8	73.53	88.86	67.6

(A) Platelet-rich plasma aggregated immediately after adding TRAP (blue) or 5 µg/ml Horm collagen (red), while no aggregates formed with 5 µg/ml collagen G (black). At 100 µg/ml, collagen G induced platelet aggregation (wine), but with a much longer lag time compared to 5 µg/ml Horm collagen. 100 µg/ml PLL (green) did not induce aggregation.

(B) Washed platelets aggregated immediately after adding TRAP (blue), or 5 µg/ml Horm collagen (red). Collagen G did not induce aggregation of washed platelets at 5 µg/ml (black), but induced aggregates at 100 µg/ml (wine). PLL (100 µg/ml) induced aggregation of washed platelets (green) but with much shorter lag phase and in a different way than TRAP and collagens did. The table shows detail data points for maximum aggregation (%), speed/slope of aggregation (%/min), and lag phase (min).

Supplementary Figure 3. Confocal laser scanning microscopy images of platelets loaded with Ca^{2+} indicator Fluo4-AM.

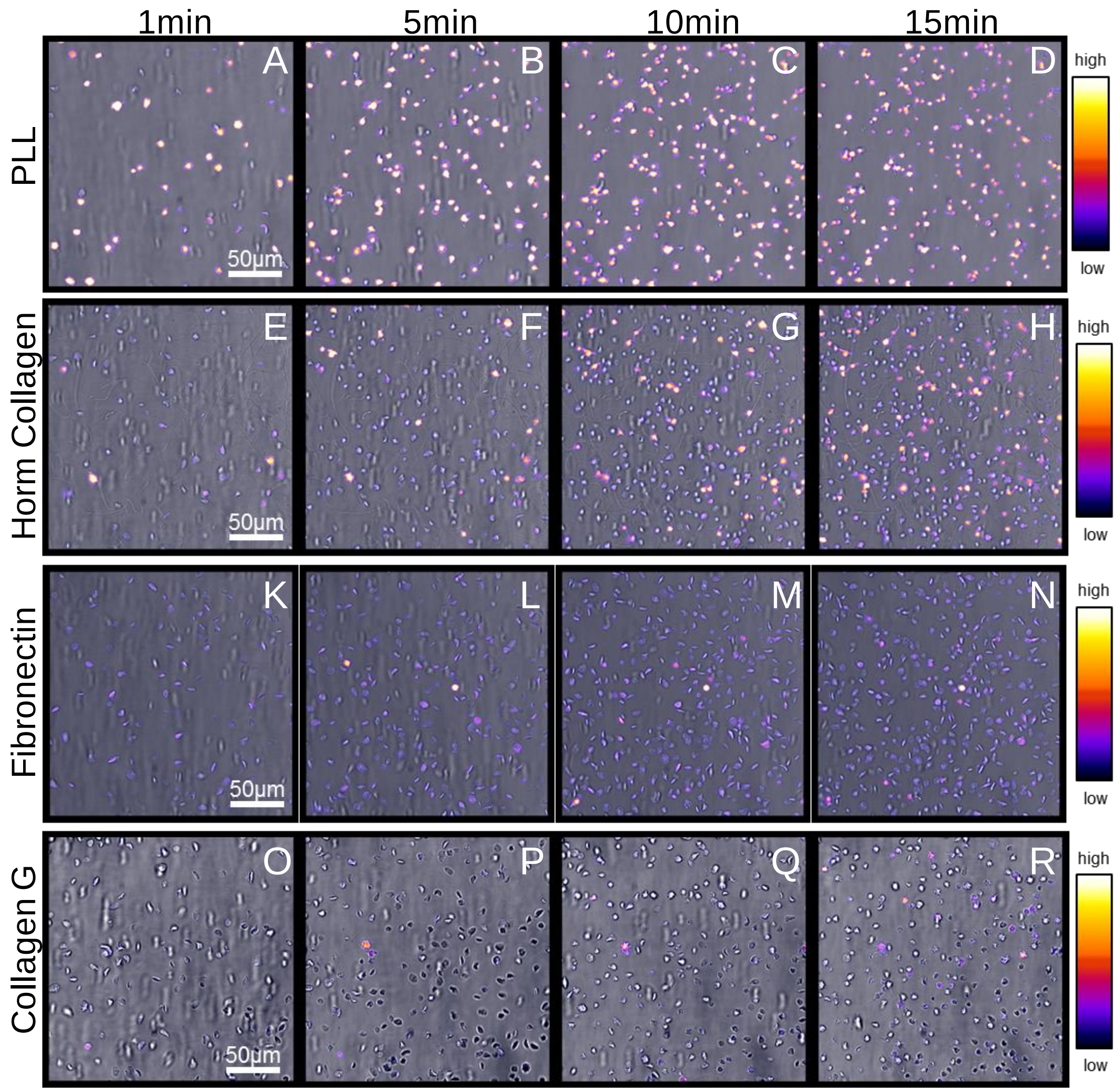

At any time point, highest calcium signaling level was from platelets on PLL (**A-D**), followed by Horm collagen (**E-H**), lower on fibronectin (**K-N**), and lowest on collagen G (**O-R**). Higher calcium signaling level indicates more activation and spreading of platelets.

Supplementary Figure 4. Comparison of force- and work- histogram distributions obtained when platelets were immobilized on PLL.

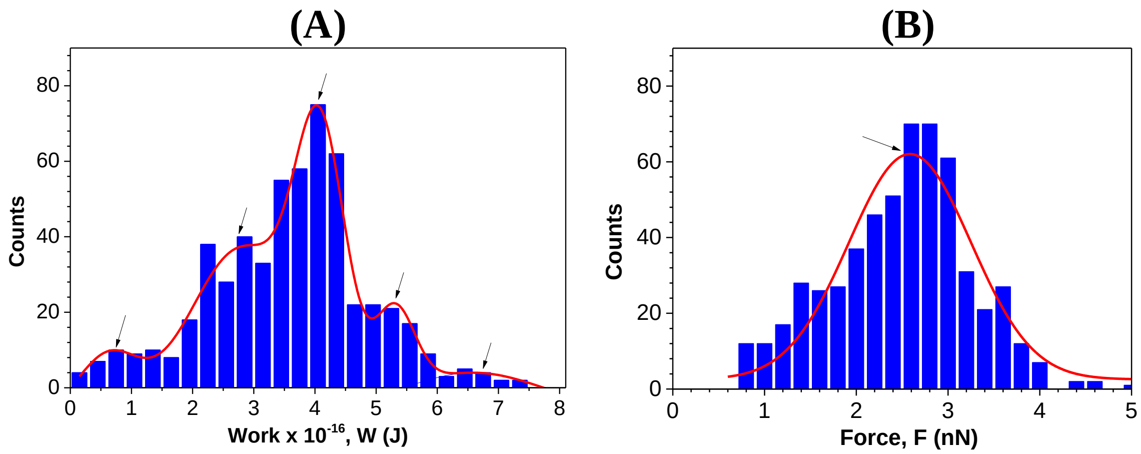

Multiple peaks (black arrows) can be clearly observed in the work histogram distribution (**A**), but not in the force histogram distribution (**B**).

Supplementary Figure 5. Characteristics of material-passivated substrates.

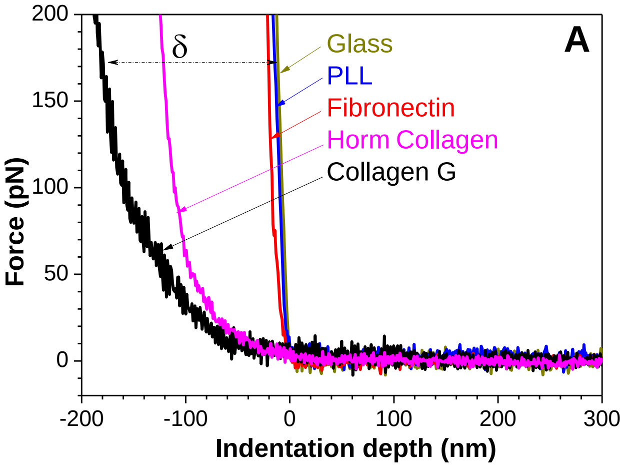

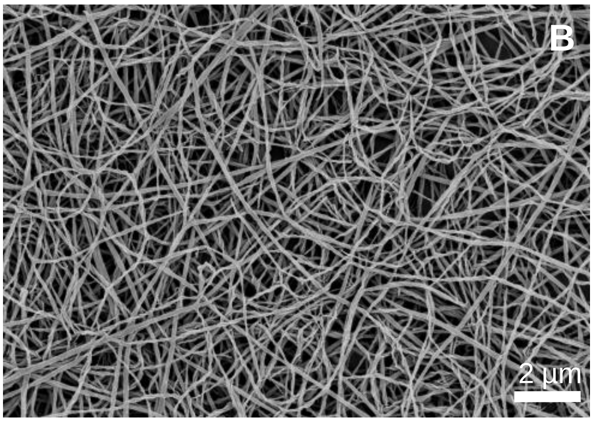

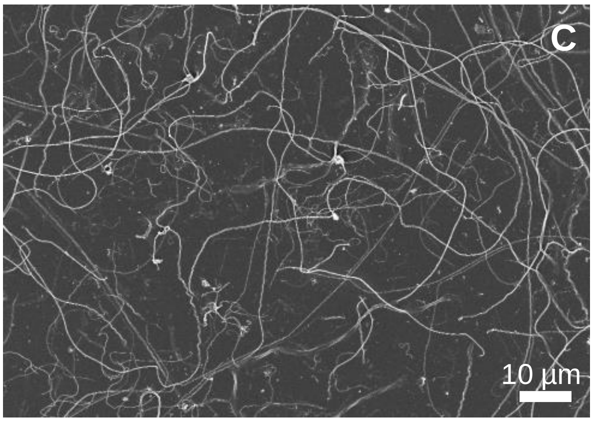

Stiffness of material-passivated surfaces measured by force-indentation method using a silica colloid probe. (**A**) Glass substrate (**dark yellow**) was used as a reference hard material for determination of the indentation depth (σ) formed by other soft materials. No significant indentation depth was observed on PLL (**blue**) and fibronectin (**red**). Indentation depth was deeper on Horm collagen (**magenta**), and deepest on collagen G (**black**). Deeper indentation depth indicates that a thicker gel was formed on the substrate. The Young's modulus of the gel formed by collagen G was lower (E = 1.561 ± 0.013 kPa) than that of Horm collagen (E = 2.315 ± 0.122 kPa). The results indicate that a thick, but soft gel was formed by collagen G, a thinner and stiffer gel formed by Horm collagen, and much thinner layers were formed by fibronectin and PLL. SEM images also show that multiple layers were formed by collagen G (**B**) and thinner layers were formed by Horm collagen (**C**). Multiple layers formed by collagen G induced higher noise in the force-indentation curve as indicate by larger variation when the colloid probe was in contact with this layer. Horm collagen comprises fibers with different sizes, while collagen G consists of more uniform fibers.

Supplementary Movie 1: Confocal laser scanning microscopy movie of platelets loaded with Ca^{2+} indicator Fluo4-AM on PLL.

Supplementary Movie 2: Confocal laser scanning microscopy movie of platelets loaded with Ca^{2+} indicator Fluo4-AM on Horm collagen.

Supplementary Movie 3: Confocal laser scanning microscopy movie of platelets loaded with Ca^{2+} indicator Fluo4-AM on fibronectin.

Supplementary Movie 4: Confocal laser scanning microscopy movie of platelets loaded with Ca^{2+} indicator Fluo4-AM on collagen G.
